# The Gpr1-regulated Sur7 family protein Sfp2 is required for hyphal growth and cell wall stability in the mycoparasite *Trichoderma atroviride*

**DOI:** 10.1038/s41598-018-30500-y

**Published:** 2018-08-13

**Authors:** Lea Atanasova, Sabine Gruber, Alexander Lichius, Theresa Radebner, Leoni Abendstein, Martin Münsterkötter, Nancy Stralis-Pavese, Paweł P. Łabaj, David P. Kreil, Susanne Zeilinger

**Affiliations:** 1 Institute of Microbiology, University of Innsbruck, Innsbruck, Austria; 2 Institute of Chemical, Environmental & Bioscience Engineering, TU Wien, Vienna, Austria; 3 MIPS - Institute of Bioinformatics and Systems Biology, Helmholtz Zentrum München, Munich, Germany; 4 Functional Genomics and Bioinformatics, Sopron University, Sopron, Hungary; 5 Chair of Bioinformatics, Boku University Vienna, Vienna, Austria; 6 Present Address: Institute of Food Technology, University of Natural Resources and Life Sciences (BOKU), Vienna, Austria

## Abstract

Mycoparasites, e.g. fungi feeding on other fungi, are prominent within the genus *Trichoderma* and represent a promising alternative to chemical fungicides for plant disease control. We previously showed that the seven-transmembrane receptor Gpr1 regulates mycelial growth and asexual development and governs mycoparasitism-related processes in *Trichoderma atroviride*. We now describe the identification of genes being targeted by Gpr1 under mycoparasitic conditions. The identified gene set includes a candidate, *sfp2*, encoding a protein of the fungal-specific Sur7 superfamily, whose upregulation in *T*. *atroviride* upon interaction with a fungal prey is dependent on Gpr1. Sur7 family proteins are typical residents of membrane microdomains such as the membrane compartment of Can1 (MCC)/eisosome in yeast. We found that GFP-labeled Gpr1 and Sfp2 proteins show partly overlapping localization patterns in *T*. *atroviride* hyphae, which may point to shared functions and potential interaction during signal perception and endocytosis. Deletion of *sfp2* caused heavily altered colony morphology, defects in polarized growth, cell wall integrity and endocytosis, and significantly reduced mycoparasitic activity, whereas *sfp2* overexpression enhanced full overgrowth and killing of the prey. Transcriptional activation of a chitinase specific for hyphal growth and network formation and strong downregulation of chitin synthase-encoding genes were observed in Δ*sfp2*. Taken together, these findings imply crucial functions of Sfp2 in hyphal morphogenesis of *T*. *atroviride* and its interaction with prey fungi.

## Introduction

Fungal pathogens causing plant disease and thereby losses in natural resources pose a common problem in agriculture. The abusive application of chemical fungicides for plant disease control often renders plant-pathogenic fungi resistant and leads to a detrimental pollution of the environment^1^. A sustainable alternative to chemical fungicides is the use of antagonistic microorganisms^2^. Members of the genus *Trichoderma* (teleomorph *Hypocrea*, Ascomycota) are superior mycoparasites - fungi that can parasitize and kill other fungi - rendering these species potent biocontrol agents for plant disease control^3^.

*Trichoderma* spp. may recognize plant-pathogenic prey fungi via small molecules that are released by the pathogen already before contact. These molecules are supposed to bind *Trichoderma* receptors such as seven-transmembrane G protein-coupled receptors (GPCRs), thereby eliciting a signaling cascade that triggers downstream responses^4^. The mycoparasitic response includes enhancement of the expression of genes that encode enzymes for the biosynthesis of secondary metabolites and for cell wall lysis^5^.

In filamentous fungi, GPCRs sense pheromones, sugars, amino acids, nitrogen sources and even photons^6^. More than 50 putative GPCRs have been identified in the genome of the mycoparasite *Trichoderma atroviride* and four of these genes (*gpr1*, *gpr2*, *gpr3* and *gpr4*) were previously isolated and characterized^7,8^. Gpr1 turned out to play an important role in the antagonistic interactions of *T*. *atroviride* with prey fungi by governing mycoparasitism-related processes. *gpr1*-silenced mutants were avirulent in confrontation assays due to their inability to attach to and lyse prey hyphae^9^.

Micro- or nanodomain structures of the plasma membrane are supposed to act as organizing centers for dynamic processes such as membrane transport, polarized growth, and signal transduction by influencing the organization and dynamic association of receptors with interacting proteins^10^. The *Saccharomyces cerevisiae* plasma membrane contains different types of subdomains exhibiting a composition, structure, and biological function distinct from the surrounding membrane^11–13^. One of them is a protein-organized microdomain known as Membrane Compartment occupied by Can1 (MCC), which corresponds to inward furrows on the plasma membrane^11,14,15^. Sur7 family proteins are typical residents of such membrane microdomains that are stabilized by a complex of cytosolic proteins termed the eisosome^14,16^. The eisosomal portion of proteins assemble into filaments and curve the membrane to form the furrows^15^. Amongst others, two families of tetraspan proteins, one comprising Sur7 and its paralogs Fmp45, Pun1, and Ynl194c (Sur7 protein family), and the other including Nce102 and Fhn1, were discovered in the MCC^17–19^. Sur7-containing MCC domains are important for plasma membrane organization, sphingolipid homeostasis, and cell wall morphogenesis (for review see^15^). While *S*. *cerevisiae SUR7* deletion mutants showed reduced sporulation but had no obvious macroscopic growth phenotype^14,20^, CaSur7 of the opportunistic human pathogen *Candida albicans* promoted proper spatial organization of cell wall synthesis and plasma membrane organization as well avirulence^17,21^, and the mutants showed a clear growth phenotype resembling the one caused by the inhibition of β-glucan synthesis^22^. In the filamentous fungus *Aspergillus nidulans*, deletion of *surG* (*sur7* orthologue) did not lead to any obvious growth phenotypes^23^. Nevertheless, the yeast Sur7 paralogs Fmp45, Pun1, and YNL194C affect the response to nitrogen stress, cell wall integrity, and survival in stationary phase^15,24,25^.

Here, we describe the functional characterization of the *T*. *atroviride sfp2* gene encoding a Sur7 family protein that emerged from transcriptomics studies as being regulated by the Gpr1 receptor under mycoparasitic conditions. Phylogenetic analysis further revealed Sfp2 as an orthologue of yeast Pun1, a membrane protein that until now has not been studied in filamentous fungi. We identified Sfp2 as an important player in *T*. *atroviride* that affects hyphal growth and mycelial network formation, cell wall remodeling and stability, endocytosis, osmotic stress resistance, as well as mycoparasitism.

## Results

### Identification of prey-regulated genes whose transcription is affected by Gpr1

Prey-regulated genes transcriptionally affected by *gpr1* silencing were identified by microarray-based genome-wide comparison of gene expression in *T*. *atroviride* strain P1 (wild type; WT) and its *gpr1*-silenced mutant (*gpr1*-si) in response to interaction with the plant pathogenic prey fungus *Rhizoctonia solani*. A putative group of genes affected by Gpr1 during mycoparasitism emerged, with 645 genes being differently regulated in the response to the prey fungus in the *gpr1*-si mutant compared to the wild type strain (Table S1). Of these differently regulated genes, 80% encoded proteins with significant similarity to well-characterized fungal proteins or at least contained protein domains with known functions. About 60% showed a higher-expression response in the WT than in the mutant, i.e. were either upregulated instead of downregulated in the prey response, or were more upregulated, or were less downregulated. Of these, about 7% were coding for secreted proteins, whereas from the genes with a lower-expression response in the WT compared to the mutant, about 10% were coding for secreted proteins.

An analysis of assigned functional categories (FunCat) suggested that the most robustly over-represented processes characterizing genes that exhibited a prey response dependent on the Gpr1 receptor included specific fungal information pathways (Table S2). Specifically we found support for translation (FunCat ID 12.04), protein modification (14.07; strong support), protein targeting, sorting and translocation (14.04; strong), and protein activity regulation (18.01; strong). Further implicated categories included RNA synthesis (12.01; strong), genes involved in cellular transport (ER to Golgi transport 20.09.07.03; vesicle formation 20.09.07.25; intra Golgi transport 20.09.07.05; all strong) and metabolism such as phosphate metabolism (01.04; with moderately strong support), sugar, glucoside, polyol and carboxylate anabolism (01.05.02.04; moderate), and peptide antibiotics metabolism (01.20.37.03; supported by some analyses). All of these were implicated as over-represented amongst the genes dependent on Gpr1 in the response of *T*. *atroviride* to a fungal prey.

Amongst the genes showing a higher-expression response in the WT than in the mutant were mycoparasitism-related candidates, such as genes encoding aspartyl proteases; GprK- and PTH11-type GPCRs; non-ribosomal peptide synthases (NRPS) including the peptaibol synthetase Tex1; several predicted SSCRPs (including putative hydrophobins and a C-type lectin); GH18 chitinases; a putative GH75 chitosanase; a protein with a Carbohydrate-Binding Module (CBM) also known as LysM domain belonging to Family 50 (putative Tal1); glutathione S-transferases and a glutathione synthetase, the osmosensing-associated Tmk3 MAP kinase, and S-adenosyl methyltransferases, most prominent the methyltransferase Lae1, a key regulator of asexual development and mycoparasitism in *T*. *atroviride*^26^. In addition, we found genes involved in metabolism (encoding putative α-1,2-mannosidase (GH92), β-mannosidase (GH2), amidase, 1,4-α-glucan branching enzyme, members of GH63 and GH3), several GTP-binding and GTPase activating proteins, several subunits of the SWI-SNF chromatin remodeling complex, an orthologue of the *T*. *reesei* zink finger repressor of cellulase and xylanase expression *ace1*, as well as the *blu7* transcription factor gene involved in light response^27^, amongst the genes showing a Gpr1-dependent response (Table S1).

In total, 99 genes with a Gpr1-dependent response encoded transmembrane (TM) proteins with one to sixteen transmembrane domains. These included GprK- and PTH11-type GPCRs, iron permease proteins, as well as RTA1-domain containing proteins (a lipid-translocating exporter family in *S*. *cerevisiae*). Most of the identified candidates with four transmembrane domains were predicted to be involved in vesicle-mediated transport such as a Sur7 family protein and Sft2-like vesicle transport proteins.

### The genomic environment of gpr1

We were interested in identifying genes whose close proximity to *gpr1* on the chromosome may indicate (co)regulation or transcriptional interference. Comparative analysis of the contig region around *gpr1* orthologues in *T*. *reesei* (Tr123806), *T*. *virens* (Tv33049), and *T*. *atroviride* (Ta160995) genomes revealed a strongly conserved genetic structure in the three *Trichoderma* species (Fig. S1). However, in *T*. *virens* the respective genes were transcribed in the opposite direction compared to *T*. *atroviride* and *T*. *reesei*, indicating that a scaffold inversion had occurred during evolution.

Examining the syntenic region between bp 172758 and 259517 on *T*. *atroviride* contig 29 revealed genes encoding peptidases, MFS transporters, a sulphatase, the eliciting plant response-like protein Epl3^28^, a fungal-specific transcription factor, and a member of the fungal-specific Sur7 family (Fig. S1). A search within the gene set being differently regulated in the prey responses of the *T*. *atroviride* wild type and the *gpr1*-si mutant revealed that the transcription of the gene encoding the Sur7 family protein (further designated Sur7-family protein 2, *sfp2*) is dependent on Gpr1. The observed *sfp2* (Ta156007) mRNA levels were specifically upregulated upon prey contact in the wild type but not in the *gpr1*-si mutant (Table S1).

**Figure 1:**
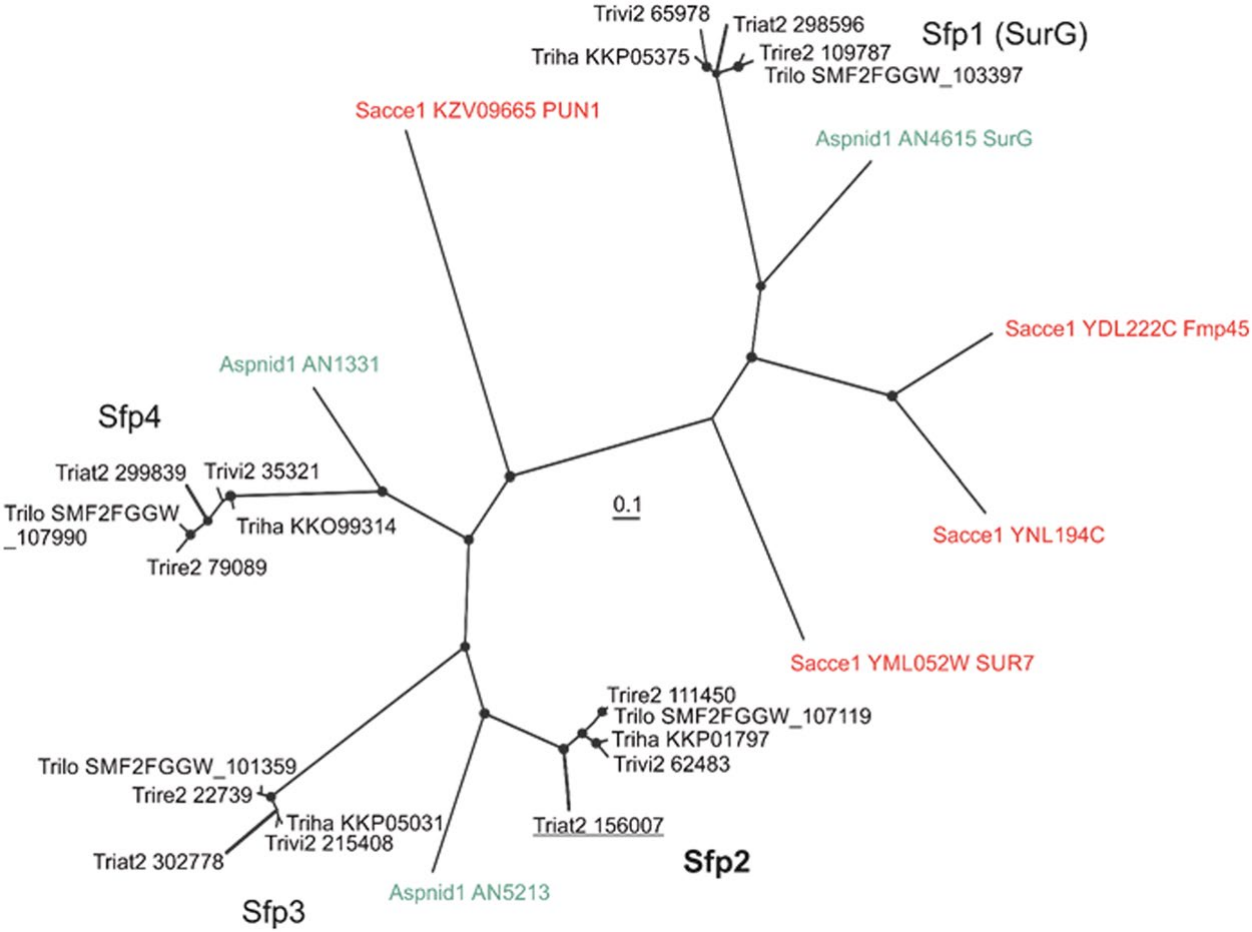
Bayesian phylogram based on amino acid sequences from Sur7 orthologues of *S*. *cerevisiae*, *A*. *nidulans* and *Trichoderma* spp. The Bayesian analysis ran for three million MCMC generations and a strict consensus tree was obtained using a Dayhoff amino acid substitution model. PP values lower than 0.95 were not considered significant and are not shown on the resulting phylogram. Sacce1, Aspnid1, Triat2, Trire2, Trilo SMF2FGGW, Trivi2 and Triha are derived from the respective available genome versions of *S*. *cerevisiae*, *A*. *nidulans* FGSC A4, *T*. *atroviride* IMI 206040, *T*. *reesei* QM 6a, *T*. *longibrachiatum* SMF2, *T*. *virens* Gv29-8 and *T*. *harzianum* T6776, respectively. The underlined sequence represents the *T*. *atroviride* Sur7 family protein Sfp2.

The *sfp2* open reading frame consists of three exons and two introns and encodes a protein of 318 amino acids with four predicted transmembrane domains and a fungal-specific Sur7 domain (pfam06687). Phylogenetic analysis of Sur7 family proteins from *S*. *cerevisiae*, *A*. *nidulans* and different *Trichoderma* species revealed four clades comprising *Trichoderma* Sur7 family members with one clade representing orthologues of *A*. *nidulans* SurG (Fig. 1). SurG is a true orthologue of *S*. *cerevisiae* Sur7^23^ for which the functions were already reported. According to the phylogram (Fig. 1), the *S*. *cerevisiae* Sur7 paralog Pun1 seems to be the common ancestor of the second supported clade, which further comprises three supported subclades. *T*. *atroviride* Sfp2 and its paralogs from other *Trichoderma* species hence are more closely related to yeast Pun1 than to Sur7.

### Sfp2 impacts hyphal growth and branching

To assess the biological role of Sfp2 in *T*. *atroviride*, *sfp2* gene deletion mutants were generated. Although all of the resulting 25 transformants showed hygromycin B-resistance, further analysis yielded only one mitotically stable mutant with homologous integration and hence deletion of the *sfp2* open reading frame. Complemented strains (RE*sfp2)* were generated by reintroducing *sfp2* under control of the constitutive *pki1* promoter (P*pki1*::*sfp2-mEGFP* construct) into a random site of the genome of the Δ*sfp2* mutant. Strains overexpressing *sfp2* (OE*sfp2*) were obtained by introducing P*pki1::sfp2* with and without C-terminal mEGFP tag into the WT background. As *sfp2* expression turned out to be governed by Gpr1, the *gpr1*-silenced mutant *gpr1* sil-8^9^ was included in the study to identify putative overlapping phenotypes resulting from *sfp2* knock-out and *gpr1* knock-down, respectively.

**Figure 2:**
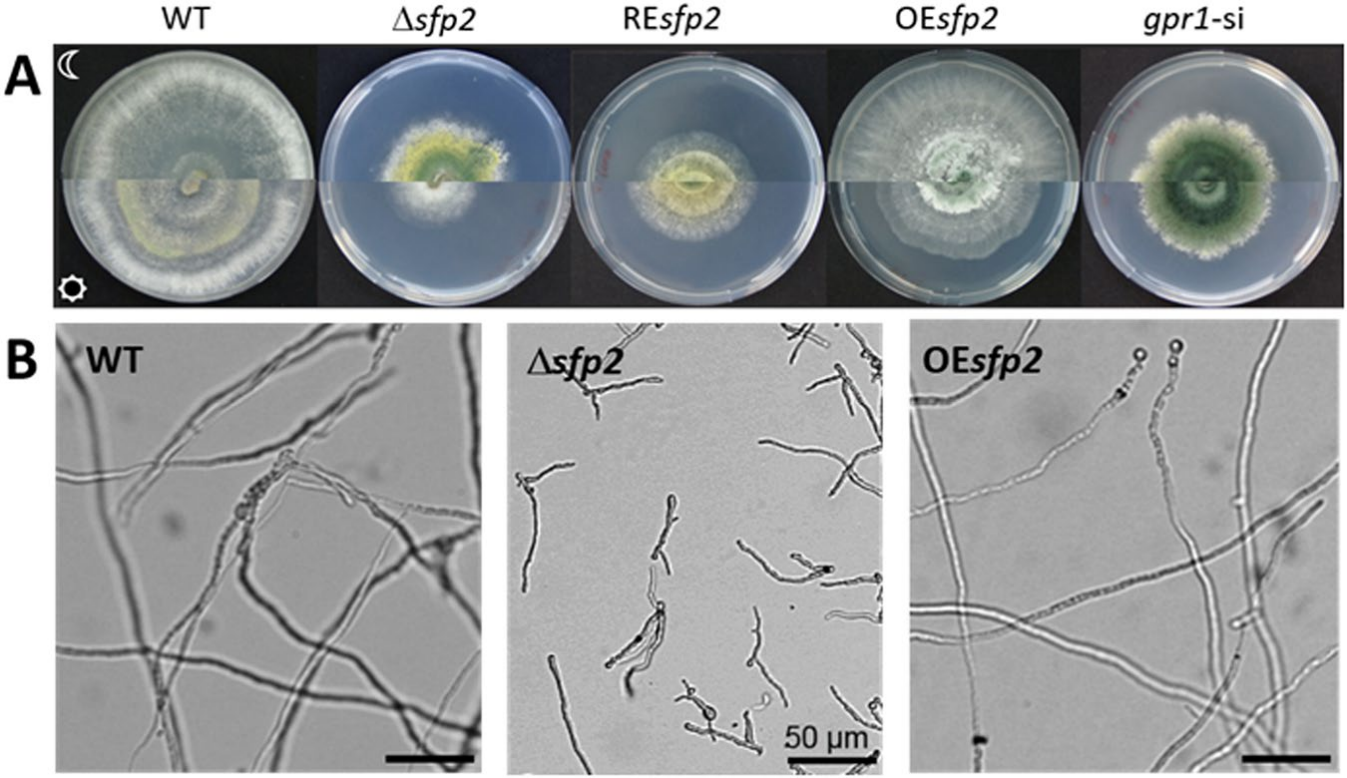
Phenotypical characterization of *T*. *atroviride sfp2* and *gpr1* mutants. (**A**) Strains grown for five days on PDA plates in complete darkness (upper part of the plates) or under cycling daylight (lower part of the plates). (**B**) Microscopic images of hyphae from *T*. *atroviride* WT, *sfp2* deletion (Δ*sfp2*) and overexpression (OE*sfp2*) mutants originating from conidia grown in PDB for 24 h. Differences in hyphal branching and germ tube lengths were observed between the strains with Δ*sfp2* exhibiting shorter and thinner germ tubes than the WT and the OE*sfp2* strain.

Striking differences in macro- and micro-morphology between the WT and the tested mutants were observed upon cultivation on complex medium agar plates in complete darkness as well as under cycling illumination. The Δ*sfp2* mutant, similar to *gpr1* sil-8, formed compact colonies with significantly less aerial hyphae than the WT, but instead exhibited invasive growth with hyphae entering the solid medium. In *T*. *atroviride*, conidia production is light-dependent^29^. Accordingly, the WT did not conidiate when cultivated in complete darkness. In contrast, Δ*sfp2* as well as *gpr1* sil-8, showed permanent, light-independent conidiation (Fig. 2A). The heavily reduced growth of the Δ*sfp2* mutant in light was partially restored by re-introducing the *sfp2* gene and the re-transformed strain still showed slight conidiation in darkness. Growth of the *sfp2* mutant strains was also monitored in liquid culture by incubating the WT and the two mutant strains, Δ*sfp2* and OE*sfp2*, in PDB for 12 h. Under these conditions, the Δ*sfp2* mutant displayed retarded germination and restricted hyphal elongation compared to the WT and the OE*sfp2* strain. After 12 h in PDB, 100% of the WT, 85% of OE*sfp2* but only 28% of the Δ*sfp2* conidia had germinated, all with unipolar germ tubes. Similar results were obtained with synthetic minimal medium. Microscopic examination of hyphae after prolonged cultivation (24 h) revealed abnormal, enhanced branching of ∆*sfp2* indicating a polarity defect caused by deletion of the *sfp2* gene (Fig. 2B).

### Sfp2 is involved in osmotic stress regulation and mycoparasitic activity

**Figure 3:**
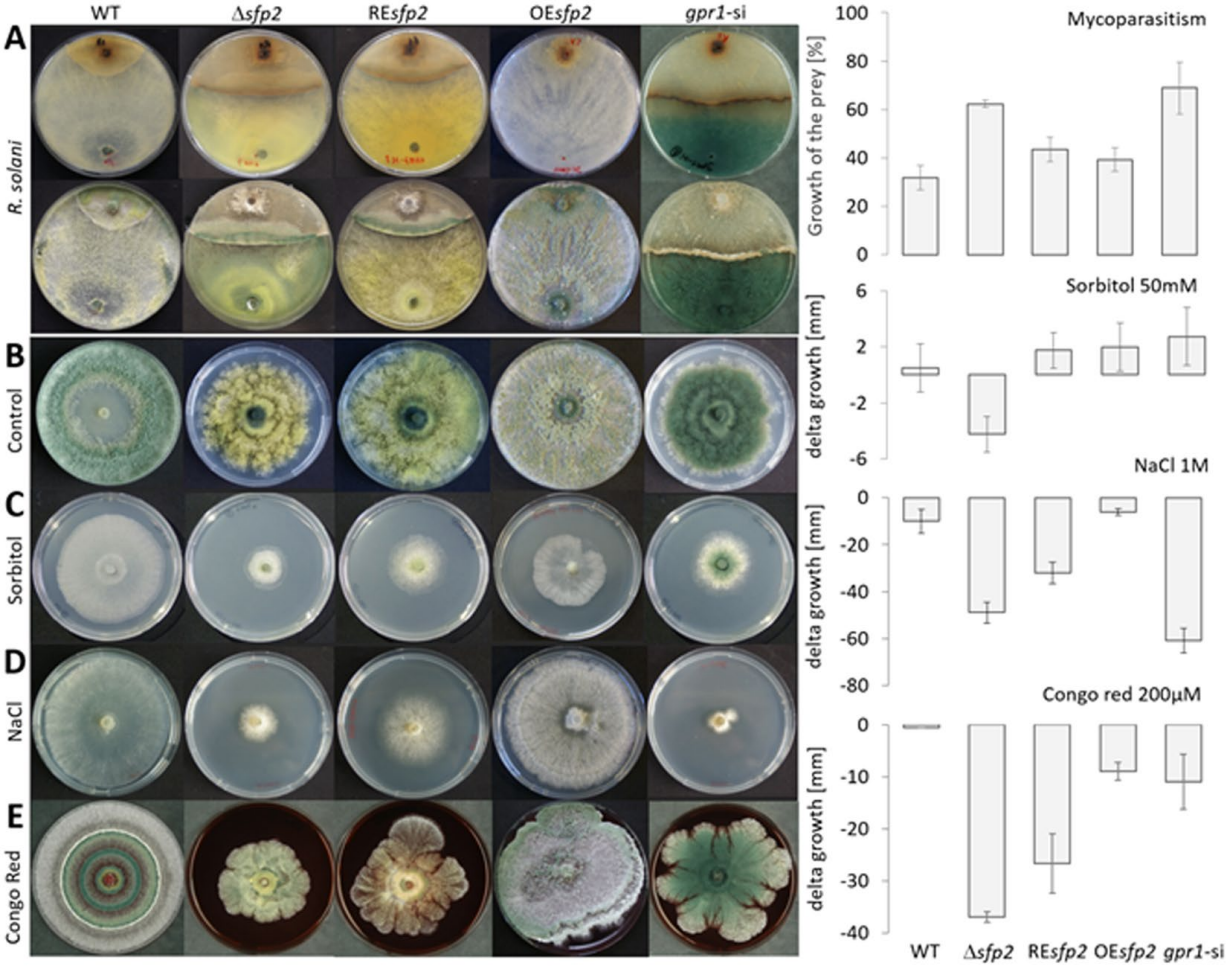
Involvement of Sfp2 in antagonism of *T*. *atroviride* against *R*. *solani* and in the response to salt, osmotic and cell wall stress. (**A**) Dual confrontation assays against *R*. *solani* (back and front sides of the plates are shown). (**B**) Cultures of *T*. *atroviride* strains on PDA without any stressor. Growth on PDA supplemented with 50 mM sorbitol (**C**), 1 M NaCl (**D**) and 200 µM Congo Red (**E**). Plates were incubated in darkness at 25 °C for seven days. Graphs represent the antagonistic potential and stress sensitivity of the different *T*. *atroviride* strains. Error bars annotate the standard deviations based on at least three biological replicates.

Our previous analyses of *gpr1*-silenced mutants revealed a complete loss of mycoparasitic activity, i.e. inability of the mutants to attach to, overgrow and lyse prey hyphae^9^. To test whether *sfp2* is involved in mycoparasitism, plate confrontation assays against *R*. *solani* as fungal prey were performed. Compared to the WT that attacked and overgrew the prey within seven days, the ∆*sfp2* mutant had a reduced antagonistic potential evidenced by a lower growth inhibition of *R*. *solani*. The ability to suppress the growth of the fungal prey slightly recovered in the genetically complemented Re*sfp2* mutant, while overexpression of *sfp2* led to a very strong mycoparasitic response with enhanced overgrowth and killing of the prey (Fig. 3A).

Exposure to sorbitol-mediated osmotic stress and sodium chloride-mediated salt stress resulted in strongly impaired growth of ∆*sfp2* (Fig. 3B). Interestingly, the *gpr1*-si mutant tolerated osmotic stress better than ∆*sfp2*, but was strongly susceptible to salt stress. Both, salt as well as osmotic stress, however, only slightly altered the growth rate of OE*sfp2* and the WT (Fig. 3C,D).

### Sfp2 is required for cell wall stability and endocytosis

To test cell wall stability, strains were grown on PDA supplemented with 200 µM Congo Red (CR), a dye that forms a complex with (helical) chain parts of chitin networks and results in a loss of cell wall rigidity due to compromised lateral interaction between the helices^30^. CR strongly affected ∆*sfp2* causing a 43% growth reduction compared to the control condition (PDA only), whereas growth of the WT and the *gpr1*-si mutant was not significantly altered by the addition of the dye (Fig. 3B).

**Figure 4:**
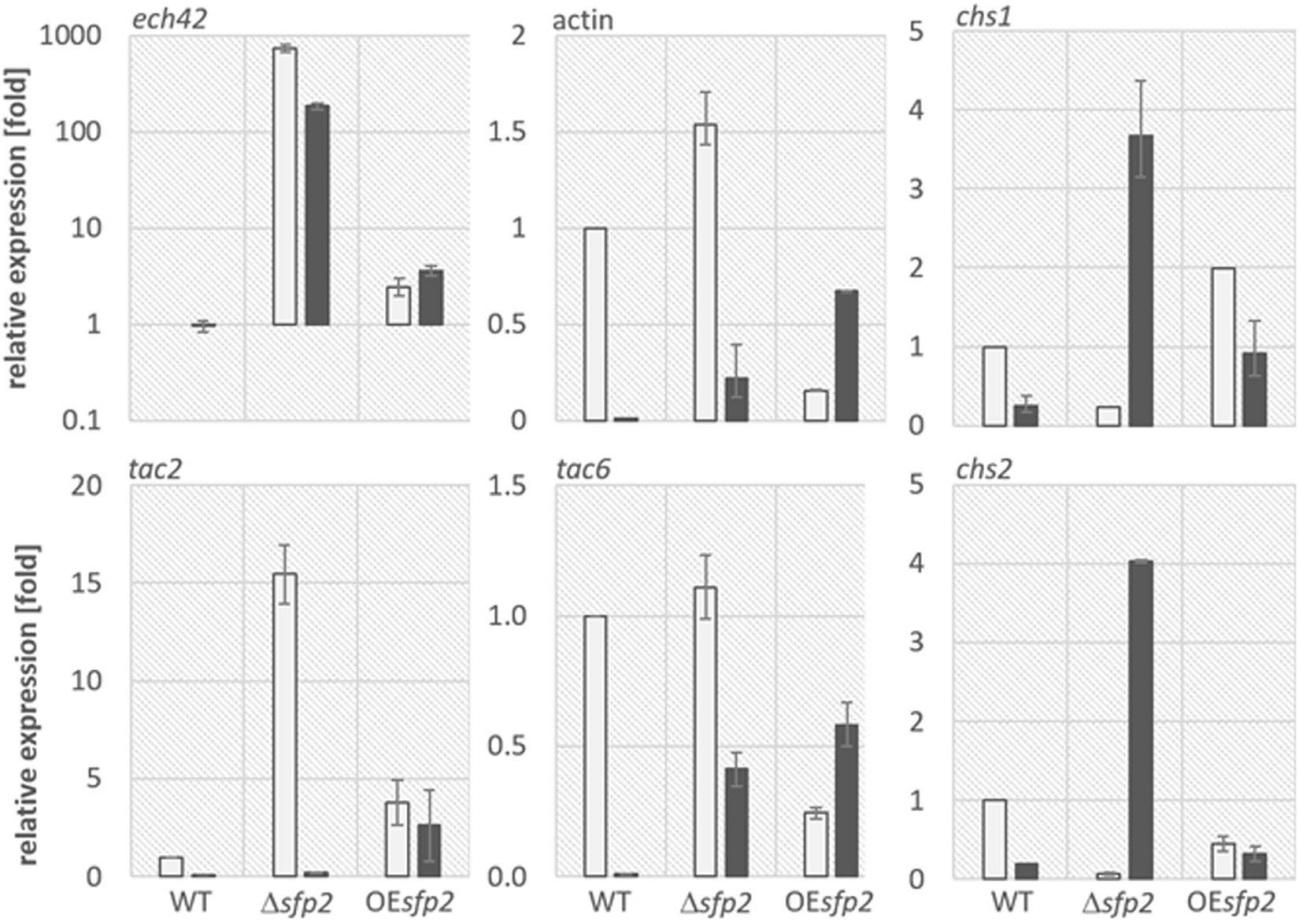
Transcriptional analysis of chitinase, actin and chitin synthase genes in *T*. *atroviride* during self-confrontation (white bars) and confrontation with *R*. *solani* (black bars, sampling at the hyphal contact stage) in WT, *sfp2* deletion and overexpression mutants. Transcript patterns of sgC chitinase genes *tac2* and *tac6*, chitinase *ech42*^31^, two chitin synthases, *chs1* (Ta142365) with myosin domain and class I *chs2* (Ta323101), as well as actin gene (Ta297070) were analyzed to elucidate the effect of *sfp2* deletion on the regulation of cell wall remodeling activities. *sar1* was used as a reference gene^7^. The error bars represent standard deviation based on at least three sample replicates.

We next tested the expression of genes putatively involved in the remodeling, repair and degradation of the fungal cell wall, such as chitinases and chitin synthases, when *T*. *atroviride* was confronted with itself (control) or *R*. *solani* (mycoparasitism). Subgroup C chitinases encoded by *tac2* and *tac6* are responsible for hyphal growth and network formation of *T*. *atroviride* but not for mycoparasitism^31,32^. In our study, transcription of *tac2* and *tac6* was strongly downregulated in the WT but also in ∆*sfp2* when confronted with *R*. *solani*. Upon contact with its own hyphae, however, ∆*sfp2* induced the expression of *tac2* more than 15 fold (Fig. 4). These results indicate that *sfp2* might regulate the machinery for self-cell-wall hydrolysis and thereby control growth and network formation of *T*. *atroviride* hyphae. Elevated *tac2* transcription levels in the ∆*sfp2* mutant could be due to its increased hyphal branching (Fig. 2B). However, when grown in shaking liquid medium (reduced self-contact) the expression of *tac2* was reduced in ∆*sfp2* compared to the WT. The addition of CR into the liquid medium, however, induced *tac2* transcription in ∆*sfp2* and led to reduced *tac2* mRNA levels in OE*sfp2* compared to the WT and compared to growth without CR (Fig. S2). We assume that the overexpression of *sfp2* likely causes increased cell wall stability that better endures the loss of rigidity due to cell wall intercalation and consequently reduces expression of *tac2*. Surprisingly, the expression of *tac6* was not affected in *sfp2* mutants during self-confrontation. Transcription of *ech42* (encoding endochitinase 42 that is the most abundant *Trichoderma* chitinase) was massively induced in ∆*sfp2*, particularly when confronted with itself but also in contact with *R*. *solani*. In liquid cultures, *ech42* was constitutively expressed in all *sfp2* mutant strains and the WT.

**Figure 5:**
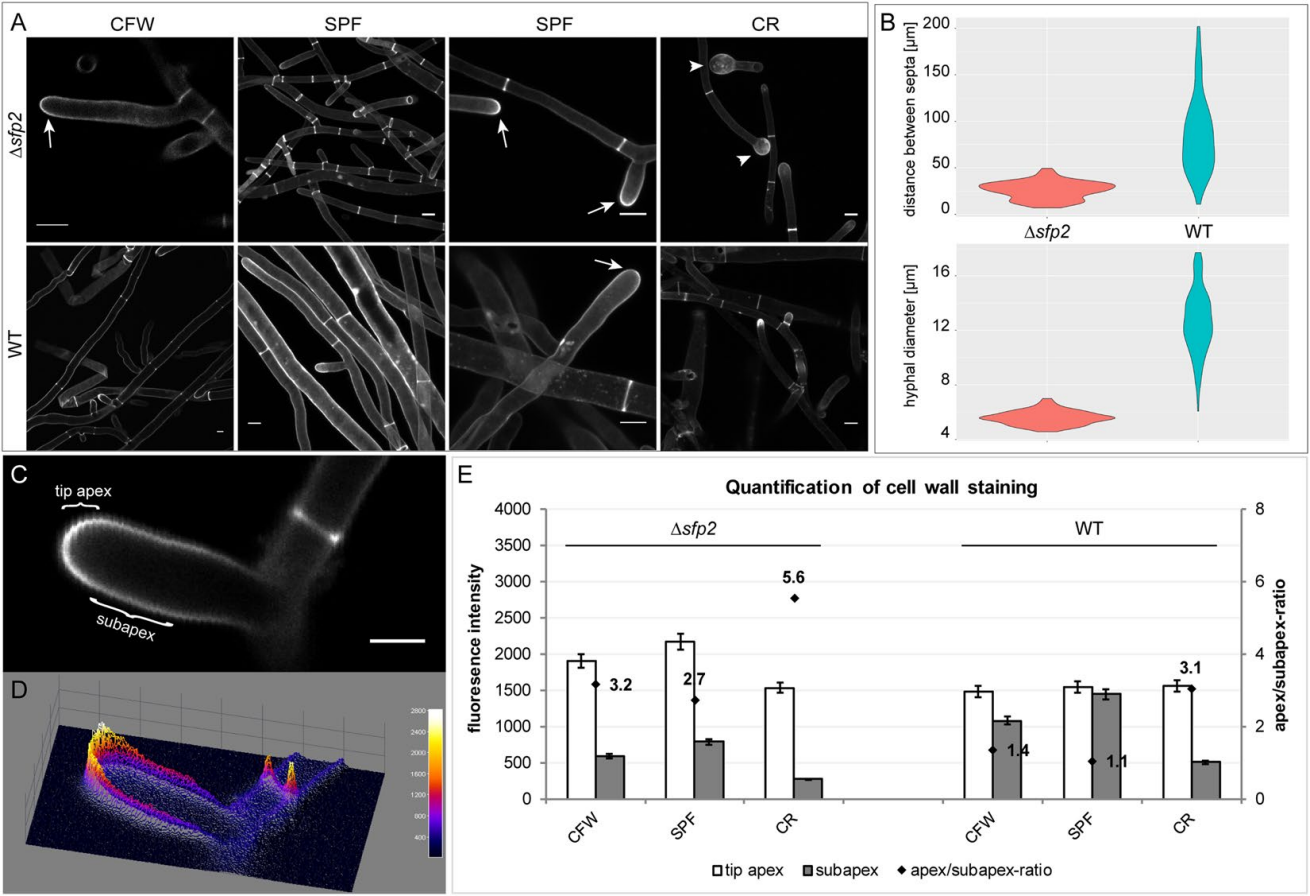
Deletion of *sfp2* changes the deposition pattern of cell wall material and affects hyphal morphogenesis (**A**) Calcofluor White (CFW) and Solophenyl Flavine (SPF) are non-specific stains of β-1,4-glucans, including chitin, and indicate that more cell wall polymers are deposited at the tip apices of Δ*sfp2* hyphae (arrows) compared to the WT. Congo Red (CR), regarded as a specific stain of α- and β-chitins, disrupts polarized tip extension much more effectively in Δ*sfp2* than in the WT, leading to extensive tip swelling of mutant hyphae (arrowheads). Scale bars, 10 µm. (**B**) The morphogenetic defects caused by *sfp2* deletion become obvious by the significantly reduced average distance between septa (Δ*sfp2* = 26.0 µm, WT = 85 µm; n = 60; ANOVA Pr < 2^−16^) and the much smaller average hyphal diameter (∆*sfp2* = 5.6 µm, WT = 12.6 µm; n = 100; ANOVA Pr < 2^−16^). (**C**) Imaging example showing increased dye fluorescence in the tip apex compared to the subapical cell wall region. Scale bar, 5 µm. (**D**) 3D surface plot of (**C**) showing the relative distribution of dye incorporation as colour-coded intensity map. (**E**) Quantification of the relative fluorescence intensities of cell wall staining in Δ*sfp2* mutant compared to the WT (n = 55).

Chitin synthases (CHS) are essential for hyphal growth and are supposed to be involved in the repair of the fungal cell wall^33–35^. Decreased transcriptional levels of two chitin synthase-encoding genes, *chs1* (encoding a CHS with a myosin domain) and *chs2* (encoding a class I CHS), were evident in ∆*sfp2* upon self-confrontation (Fig. 4). However, upon confrontation with *R*. *solani* transcription of both chitin synthase genes was significantly upregulated in the Δ*sfp2* mutant (up to 4-fold), whereas it was downregulated in the WT and in the *sfp2* overexpression mutant compared to non-antagonistic growth conditions. In addition, actin, that plays an important role in forming microfilaments^36^, was transcriptionally upregulated in Δ*sfp2* upon self-contact, whereas in OE*sfp2* its transcription was significantly downregulated relative to the WT (Fig. 4). These results indicate that cell wall integrity, and consequently cell wall synthesis and autolysis, are likely dependent on Sfp2. This is reflected in the ∆*sfp2* growth phenotype that lacks hyphal diversification, i.e. all hyphae in the periphery and subperiphery of the colony looked similar and did not differentiate into leading hyphae and primary and secondary lateral branches. We further tested this hypothesis by staining the cell wall with various dyes selective for β-1,4-glucans, including chitin, thereby revealing some additional features of the ∆*sfp2* hyphal growth phenotype (Fig. 5). Hyphae of Δ*sfp2* showed a pronounced deposition of chitin at tip apices compared to the WT and furthermore, displayed increased sensitivity to CR which led to extensive isotropic tip swelling (arrows and arrowheads in Fig. 5A). In addition, the average distance between septa (Δ*sfp2* = 26.0 µm, WT = 85 µm; n = 60; ANOVA Pr < 2^−16^) and the average hyphal diameter (∆*sfp2* = 5.6 µm, WT = 12.6 µm; n = 100; ANOVA Pr < 2^−16^) in the mutant were significantly reduced when compared to the WT (Fig. 5A,B). Quantification of the ratio of the relative dye fluorescence between tip apex and subapex revealed interesting changes in the cell wall deposition pattern between Δ*sfp2* and the WT (Fig. 5C–E). In comparison to the WT, the absolute amount of CFW and SPF dye incorporated at the tip apices of the mutant was elevated (CR was equal), whereas the signal intensity of all three dyes in the subapex was much reduced. Consequently, the ratio between apical and subapical fluorescence of all dyes in the mutant was significantly higher compared to the WT. This indicates pronounced cell wall deposition at growing hyphal tips but weaker consolidation of cell wall structure in the subapex of Δ*sfp2* hyphae. Together, these features support the notion of a highly disturbed and chemically altered cell wall organization in the ∆*sfp2* mutant, leading to a fundamentally de-regulated control over hyphal morphogenesis. Another hint for this might be a possible preference of two distinct septal distances of about 15 and 30 µm in ∆*sfp2* that can be observed as slightly bimodal shape of the violin plot of ∆*sfp2* septal distance (Fig. 5B).

**Figure 6:**
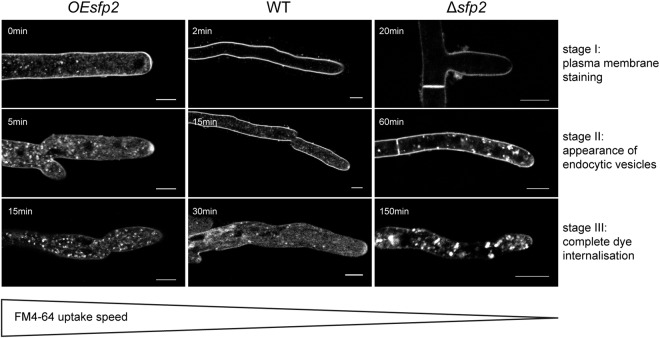
Influence of Sfp2 on the endocytic uptake of FM4-64. The figure shows three successive key stages of the dye uptake process that are easily discernible in the WT. Stage I: exclusive plasma membrane staining (top row), stage II: first appearance of the dye in endocytic vesicles (middle row), and stage III: exclusive staining of endocytic vesicles and endomembranes (bottom row). Equivalent staining patterns are shown at the earliest time point of their appearance in the respective strain. In comparison to the wild type (WT), endocytosis is slightly accelerated in the *sfp2* over-expressing mutant (OE*sfp2*), whereas dye uptake is dramatically delayed in the gene deletion mutant (Δ*sfp2*). For instance, dye uptake into the plasma membrane occurs instantly in OEsfp2 but takes two minutes in the WT; and complete internalisation of FM4-64 dye from the plasma membrane occurs 10x faster in OE*sfp2* compared to Δ*sfp2*. Scale bars, 5 µm.

Sur7 family proteins are typical residents of membrane microdomains such as the MCC/eisosome in yeasts^15^. We therefore tested the effect of Sfp2 on endocytosis using the membrane-selective FM4-64 dye as marker for endosome internalization (Fig. 6). While OE*sfp2* showed slightly accelerated dye uptake compared to the WT, Δ*sfp2* displayed significantly delayed endocytosis, obvious by the first appearance of intracellular dye molecules only after 45–60 min post FM4-64 addition. An equivalent staining pattern occurred in the WT already after 10–15 min.

### Gpr1 and Sfp2 show overlapping but not identical localization patterns

**Figure 7:**
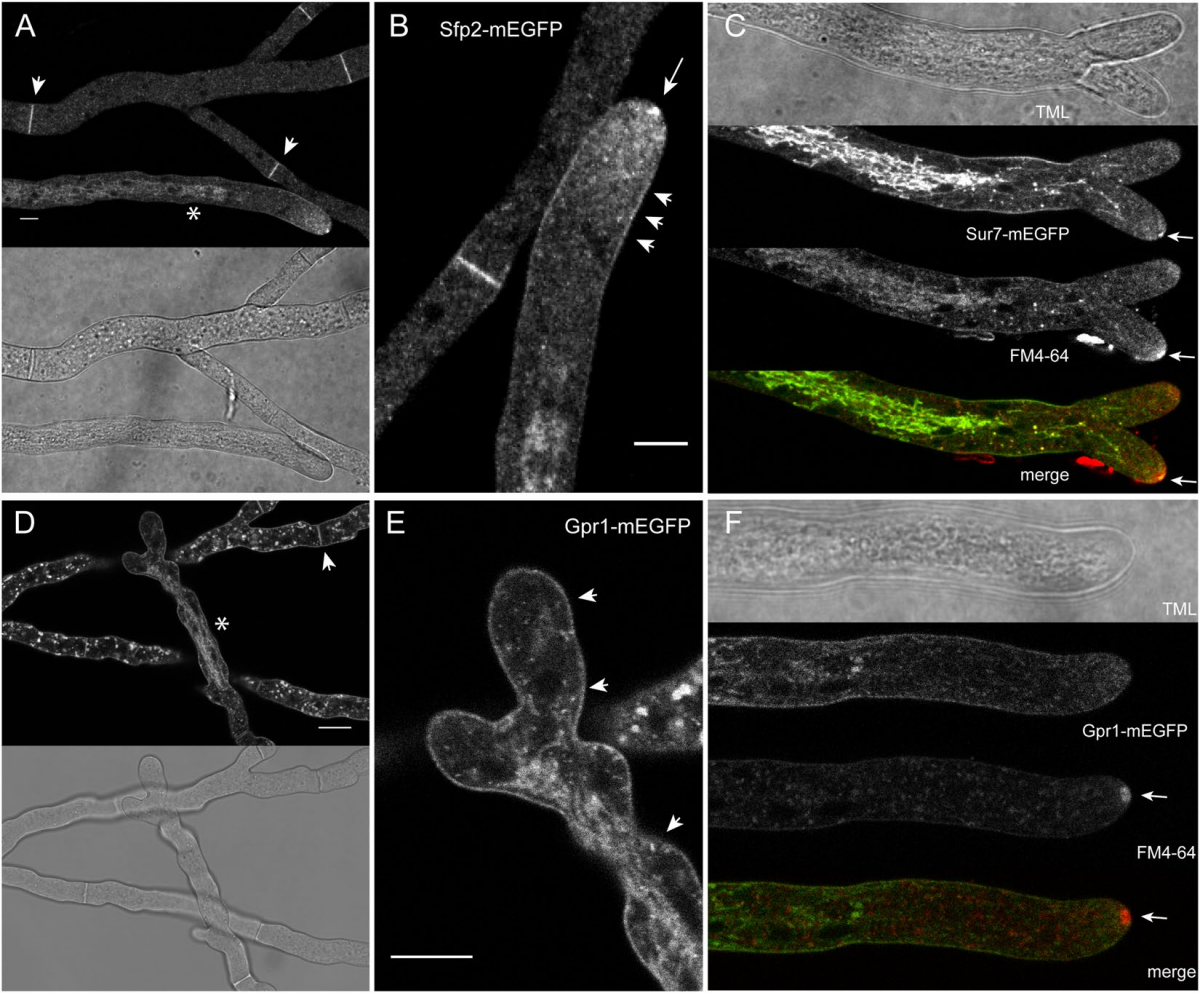
Sfp2-EGFP and Gpr1-EGFP show partially overlapping but not identical subcellular localizations associated to the exo- and endocytic pathways. (**A**) Sfp2-mEGFP decorates the plasma membrane, septa (arrowheads), and tubular vacuoles (asterisks). (**B**) Magnification of hyphal tip from (**A**) showing that besides the plasma membrane (arrowheads), Sfp2-mEGFP is also part of the Spitzenkörper (arrow) and intensely clusters at the position of the subapical endocytic ring (arrowheads). (**C**) Co-staining with FM4-64 proves that Sfp2 is part of the Spitzenkörper. (**D**) Gpr1-mEGFP as well localizes to the plasma membrane, septa (arrowheads), and numerous internal membrane systems suspected to include tubular vacuoles (asterisks) and additional compartments, most probably part of the endocytic pathway, that are not obviously occupied by Sfp2. (**E**) Magnification of the hyphal tip from (**D**) highlighting widespread plasma membrane decoration of Gpr1-mEGFP essentially covering the whole hyphal surface (arrowheads). (**F**) Co-staining with FM4-64 proves that Gpr1 is not part of the Spitzenkörper. Scale bars, 10 µm.

Life-cell imaging analyses of *T*. *atroviride* transformants expressing Gpr1-mEGFP and Sfp2-mEGFP, respectively, showed that both proteins have similar, greatly overlapping but not fully identical, subcellular localization patterns (Fig. 7). As expected, Gpr1 and Sfp2 both reside at the plasma membrane, including highly pronounced accumulation at septa, and are associated to intracellular membrane clusters and vesicles, presumably associated to the endocytic pathway. Both proteins furthermore localize to what appear to be tubular vacuoles in the subapical compartment of leading hyphae. Most notably, Sfp2 is – in contrast to Gpr1 – also part of the Spitzenkörper (arrows in Fig. 7B,C) and clusters at the position of the subapical endocytic ring (arrowheads in Fig. 7B). These subcellular localizations strongly suggest that Sfp2 is part of the exo-/endocytic machinery driving polarized tip growth, which is in line with the observed endocytosis defect of Δ*sfp2*.

## Discussion

Mycoparasitism is a trait prominent within the genus *Trichoderma*. Signaling via heterotrimeric G proteins plays a major role in regulating mycoparasitism-related functions^4^ and in *T*. *atroviride*, the seven-transmembrane receptor Gpr1 was found to be essential for the antagonistic interaction of the mycoparasite with prey fungi^9^ as well as for vegetative growth, conidiation and conidial germination^7^. The g*pr1-*si mutants were unable to respond to the presence of a living prey fungus; consequently, prey hyphae remained intact and undamaged upon confrontation with the mutants^9^. We compared the gene expression response of the *gpr1*-si mutant and its parental strain upon contact with the prey *R*. *solani*. The top ten genes that were most robustly identified as differently regulated encode proteins involved in protein synthesis and cell detoxification (Table S1), including a putative RTA1-like protein whose family comprises fungal proteins with multiple transmembrane regions that may be involved in the efflux of xenobiotics^37^; a putative mitochondrial endoribonuclease of the isochorismatase superfamily presumably involved in processing and turnover of mitochondrial RNAs; translation elongation factor 2; a putative monooxygenase involved in ubiquinone biosynthesis; a glutathione S-transferase catalyzing the conjugation of the reduced form of glutathione to xenobiotic substrates for the purpose of detoxification^38^; a GH3 family protein; a mitochondrial/plastidial beta-ketoacyl-carrier protein reductase; and two proteins with unknown function. Beta-ketoacyl-ACP reductase is a highly conserved enzyme catalyzing the second step of the mitochondrial fatty acid synthesis pathway. The importance of this pathway has recently been recognized in humans, as it is related to lipoic acid synthesis diseases such as mitochondrial respirational dysfunction and accelerated aging^39^. Furthermore, the response of the plant-pathogenic prey to *Trichoderma* attack likely results in secretion of secondary metabolites and reactive oxygen species (ROS) that activate *Trichoderma* genes involved in stress response and detoxification such as reductases and MFS transporters. Several such mycoparasitism-relevant genes were differently regulated in the prey responses of WT and mutants, including aspartyl proteases, protein kinases, nonribosomal peptide synthases (NRPSs), methyl- and acetyltransferases, heat shock proteins (HSP), glutathione S-transferases, glutathione synthetase, small secreted cysteine rich proteins (SSCRP), chitinases (GH18), glycoside hydrolase family 16 (GH16) proteins, N-acetylglucosamine transferases, MFS superfamily members, and carbon binding module 50/LysM domain (CBM50) proteins. These results are in accordance with the current model of mycoparasitism where *Trichoderma* spp. recognize a prey fungus via small molecules (e.g. peptides) that are released from the prey upon the action of secreted *Trichoderma* hydrolases^5^. The released molecules may bind to *Trichoderma* cell surface receptors such as Gpr1, thereby eliciting a signaling cascade comprising G proteins, the cAMP pathway and mitogen-activated protein kinases (MAPKs), which may ultimately modulate the activities of transcription factors and consequently the expression of genes that encode enzymes for the biosynthesis of secondary metabolites and cell wall lysis^5^. The transcriptional response to prey of the candidate genes identified in our study depends on Gpr1, including the Tex1 NRPS, the Tmk2 and Tmk3 MAP kinases and the Lae1 methyltransferase. In *T*. *virens*, disruption of the *tex1* gene resulted in the loss of production of 18-residue peptaibols^40^, linear peptide antibiotics produced by *Trichoderma* and other fungal mycoparasites^41^. Peptaibols directly contribute to the antagonistic action of *Trichoderma* mycoparasites as they synergistically act with cell wall-degrading enzymes leading to a combined enzymatic degradation of the prey cell wall and membrane permeabilization^42,43^. The Tmk3 MAPK is part of the stress-activated protein kinase (SAPK) signaling pathway and was recently shown to be involved in regulation of osmotic and oxidative stress, cell wall damage, high temperature, cadmium, and UV irradiation in *T*. *atroviride*^44^. Tmk3 was rapidly phosphorylated upon light exposure and Tmk3 signaling was suggested to cooperate with the Blr photoreceptor complex in the activation of gene expression^44^. However, in *T*. *atroviride* blue-light is perceived through the Blue Light Regulator Complex, which in turn up-regulates a set of genes (*blu*) and down-regulates another set (*bld*), triggering asexual reproduction^27^. One of the genes, *blu7*, encoding a C2H2 zinc finger domain transcription factor, was found to be upregulated in the response of the *gpr1*-si mutant to *R*. *solani*, indicating that *blu7* expression is suppressed by Gpr1 in *T*. *atroviride* during prey contact. Indeed, Cetz-Chel *et al*.^27^ suggested that the diminished conidiation of ∆*blu7* mutants is likely a result of dysregulation of the cAMP signaling pathway and ROS production, whereas their low tolerance to continuous exposure to light indicates that Blu7 is required for adaptation supporting growth under continuous light exposure.

Among the four transmembrane protein-encoding genes differently regulated in the responses of the WT and the *gpr1*-mutant to *R*. *solani*, most of the candidates were putatively engaged in vesicle-mediated transport. This includes the Sur7 proteins, a protein with similarity to Sft2, and a putative orthologue of Der1, a component of the endoplasmic reticulum-associated degradation pathway. The Sft2 proteins might be required for the fusion of transport vesicles derived from the endocytic pathway with the Golgi complex^45^, whereas Sur7 proteins are localized at eisosomes, sites of protein and lipid endocytosis in yeast^15^. Eisosomes, at least partially, mediate this lateral plasma membrane compartmentalization, whereas Sur7-containing MCC are as well important for sphingolipid homeostasis and cell wall morphogenesis^11,14,15^. Little is known about the function of Sur7 family proteins in filamentous fungi. There are several Sur7-like proteins in *Aspergillus* species, including one strict Sur7 orthologue (SurG in *A*. *nidulans*)^23,46^. Vangelatos *et al*.^23^ showed that in *A*. *nidulans* conidiospores, but not in hyphae, SurG and two Pil proteins colocalize at the cell cortex and assemble into eisosomes late during conidial maturation. In mycelia, SurG was found to be located in vacuoles and endosomes. Deletion of the *surG* gene, however, did not lead to any obvious growth phenotype, except for moderate resistance to itraconazole. The authors suggested that conservation of eisosomal proteins within the ascomycetes is accompanied by a striking functional divergence.

In this study, we found that the gene encoding the *T*. *atroviride* Sur7 family protein Sfp2 is upregulated during the mycoparasitic interaction in a Gpr1-dependent manner. Interestingly, *sfp2* and *gpr1* are located in spatial proximity in the *Trichoderma* genomes analyzed and the surrounding genes show synteny, which may be older than the *Trichoderma* common ancestor, whereas it might not necessarily be older than the Sordariomycetes, as *Neurospora crassa* (Sordariales) bears no synteny in that region but carries *gpr1* and *sfp2* on the same supercontig. In the evolutionary distant *A*. *nidulans* (Eurotiomycetes), moreover, *gpr1* and *sfp2* orthologues are not part of the same chromosome.

Phylogenetic analysis of Sur7 family proteins from *S*. *cerevisiae*, *A*. *nidulans*, and diverse *Trichoderma* species revealed four clades comprising *Trichoderma* Sur7 family members. One clade gathered orthologues of *S*. *cerevisiae “*true” Sur7 and *A*. *nidulans* SurG whereas the other three are closely related to *S*. *cerevisiae* Sur7 family protein Pun1. As mentioned above, yeast cells lacking the MCC-localized Sur7 protein display broad defects in cellular organization and stress response^47^. We speculate that the reason for the functional discrepancy between yeasts and *A*. *nidulans*^23^ might lie in two other *S*. *cerevisiae* Sur7 paralogs, Fmp45 and YNL194C, that are more closely related to *Trichoderma* and *A*. *nidulans* SurG than the “true” ScSur7. Pun1, a previously uncharacterized MCC protein, was shown to be nitrogen-stress related, and its absence abolished filamentous growth in haploids and diploids^24^. Though Pun1 contributes to the cellular response to nitrogen stress through signaling pathways that regulate the expression of genes involved in amino acid biosynthesis^24^, *T*. *atroviride sfp2* deletion mutants did not show significantly altered biomass production upon nitrogen deprivation (data not shown). However, here we show that *sfp2* deletion results in reduced cell wall stability, increased sensitivity to osmotic and salt stress, and loss of antagonism in the mycoparasite *T*. *atroviride*.

Sur7 is needed for proper localization of actin, morphogenesis, cell wall synthesis, and the adequate response to cell wall stress in *S*. *cerevisiae*^47^. Similarly, cell wall intercalation by Congo Red strongly reduced growth of the *T*. *atroviride* ∆*sfp2* mutant, whereas the WT was not significantly affected. A massive overexpression of subgroup C chitinase-encoding gene *tac2*, responsible for hyphal network formation in *T*. *atroviride*, was further observed in the ∆*sfp2* mutant upon contact with its own hyphae, but not when grown in liquid culture. A similar result was obtained for the endochitinase-encoding gene *ech42* during self-confrontation, but also upon contact with *R*. *solani*. In accordance, e*ch42* was found to be induced in *T*. *atroviride* during growth on colloidal and fungal chitin^31^ and during mycoparasitism^48^. There is also a strong evidence that Ech42 is involved in autolysis processes in *T*. *atroviride*^49^. Moreover, the upregulation of two chitin synthases in the Δ*sfp2* mutant during mycoparasitism further underlines the activation of cell wall repair mechanisms. We also detected an increased expression of actin upon *sfp2* deletion. Actin plays an important role in morphogenesis, cytokinesis and the movement of organelles in fungal cells^30^. Many of these processes are mediated by extensive and intimate interactions of actin with cellular membranes^50^. In *C*. *albicans*, lack of *sur7* resulted in mislocalization of actin and septin, and abnormal cell wall material protruding into and forming structures within the cytoplasm^17,51^. Our cell wall staining results support the expression data and suggest pronounced apical chitin accumulation at the most actively secreting region of the fungal hyphae, the hyphal tip, whereas chitin consolidation in the most actively endocytosing region – the subapical endocytic ring^52,53^ – was reduced in the *sfp2* deletion strain. This correlates with our FM4-64 co-staining results and the dramatically delayed endocytosis rate in that mutant (further details below). Increased deposition of chitin at hyphal tips may as well be associated with the observed lack of hyphal differentiation into leading hyphae plus primary and secondary lateral branches, as well as the significantly decreased distance between septa and the reduced diameter of Δ*sfp2* hyphae as observed in this study. Both, the Δ*sfp2* and *gpr1*-si mutants form compact colonies with increased lateral branching and significantly less aerial hyphae when compared to the WT and, similar to *C*. *albicans sur7*Δ mutant^51^, Δ*sfp2* exhibits intensive invasive growth with hyphae entering the solid medium. The Δ*sfp2* mutant also displayed delayed conidial germination, restricted hyphal elongation and shorter germination tubes compared to the WT and the OE*sfp2* strain. This set of features is a typical indicator for a severe polarity defect caused by *sfp2* deletion. *C*. *albicans sur7*Δ mutants exhibited alterations of plasma membrane and cell wall organization by producing irregularly shaped hyphae with obvious intracellular invaginations^54^. The *C*. *albicans* mutants also accumulated cell wall abnormalities over time, thereby indicating defects in spatial and temporal regulation of cell wall synthesis^51^.

Micro- or nanodomain structures on the cell membrane are likely involved in receptor organization and internalization, and consequently influence GPCR signaling. The uniform distribution within the plasma membrane seems unlikely to provide sufficient enrichment to achieve the rapid responses characteristic for GPCRs. Consequently, cells probably concentrate signaling molecules in membrane microdomains^55^, regions of elevated cholesterol and glycosphingolipid content and less fluidity^56–58^. Life-cell imaging analyses of Gpr1 and Sfp2 revealed similar, partly overlapping but not identical, subcellular localization patterns. As expected, both proteins reside in or at the plasma membrane including intensive accumulation at septa, and are associated to intracellular membrane clusters and vesicles, presumably associated to the endocytic pathway as evidenced by FM4-64 co-staining. Both proteins furthermore localize to what appears to be tubular vacuoles in the subapical compartment of leading hyphae. In contrast to mitochondria, these are often densely packed in the apical compartment, which also appear as tubes or filaments^59^. Sfp2 is, in contrast to Gpr1, also part of the Spitzenkörper and appears to form a distinct subapical endocytic ring, which furthermore corroborates its likely function in the exo-/endocytic machinery driving polarized tip growth. This finding is in accordance with the polarized tip growth and endocytosis defect observed in the *sfp2* deletion strain. Given the phenotypes described here and the interplay between Gpr1 and Sfp2, we are further investigating whether there is a direct interaction between Gpr1 and Sfp2, and how both proteins dynamically localize in the *T*. *atroviride* plasma membrane during mycoparasitism.

## Methods

### Strains and culture conditions

*T*. *atroviride* strain P1 (ATCC 74058) was used in this study referred to as the wild type (WT). All fungal mutants were derived from this strain including the *gpr1*-silenced mutant *gpr1* sil-8^9^. Confrontation assays were performed using *Rhizoctonia solani* (teleomorph *Thanatephorus*, Basidiomycota) as a fungal prey. Fungal strains were cultivated and maintained on potato dextrose agar (PDA, Sigma) at 25 °C in darkness unless otherwise stated. *Escherichia coli* strains JM109 and Stellar (Clontech, TaKaRa) were used for plasmid constructions and amplification. 20 mm^2^ mycelial plugs were inoculated on PDA supplemented with 1 M NaCl and 50 mM sorbitol for analyses of osmotic stress resistance and 200 µM Congo Red to test the response to cell wall intercalation. At least three biological replicates per strain were incubated at 25 °C in darkness and culture development was measured daily. The average growth yield (delta growth) was calculated by subtracting the average growth of the strain cultivated with the stress inducer from the average growth of the strain under control conditions (PDA only). For gene expression analyses, *T*. *atroviride* mycelium from dual confrontation assays against itself or against *R*. *solani* was sampled upon fungal contact. For evaluation of gene expression during cell wall intercalation, PDB (potato dextrose broth, Sigma) with and without 5 μg/ml Congo Red was inoculated with mycelial plugs and incubated for five days at 150 rpm and 25 °C in darkness.

For transcriptome analyses, confrontation assays of *T*. *atroviride* WT and its *gpr1-* silenced mutant against *R*. *solani* or against itself (control) were performed on PDA plates covered with a cellophane membrane. Plates were incubated at 25 °C until the mycelia of both fungi made first contact. Peripheral hyphal zones of *T*. *atroviride* from at least six independent plates were sampled and shock frozen in liquid nitrogen. The universal reference sample was obtained by growing *T*. *atroviride* WT under different conditions (PDA and PDB; liquid synthetic media containing 1% glycerol, 1% N-acetylglucosamine, 1% chitin, 1% glucose, or 1% lactose as previously described^60^; in confrontation with *R*. *solani*) for 24, 48 and 72 hours and pooling equal amounts of isolated total RNA. Total RNA was isolated using the PeqGOLD TriFast DNA/RNA/Protein Purification Reagent (PEQLAB Biotechnology, VWR) followed by a further purification step with the RNeasy MiniElute Cleanup Kit (Qiagen, Valencia, CA, USA). The integrity of RNA samples was checked using an Agilent 2100 Bioanalyzer (Agilent, Santa Clara, CA, USA) and a Nanodrop ND-1000 spectrophotometer (Nano-Drop, Wilmington, Germany).

### Microarray data analysis

We exploited a custom high-density microarray platform for genome-wide transcriptional profiling of the 11,863 genes listed in the Gene Catalogue version 2 of the *T*. *atroviride* genome database. Probes were designed to be specific^61^ using the full *T*. *atroviride* genomic sequence [http://genome.jgi.doe.gov/Triat2/Triat2.info.html] (accessed June 2017), while also using all published sequences from the prey fungus *R*. *solani* as antagonistic templates to avoid unwanted cross-talk [https://genome.jgi.doe.gov/Rhiso1/Rhiso1.home.html].

Labeling and hybridization were performed with the Two-color Microarray-Based Gene Expression Analysis-Quick Amp Labelling with Tecan HS Pro Hybridization protocol (V 5.7, May 2008, Agilent Santa Clara, CA). In brief, double-stranded cDNA was synthesized from 50 ng of total RNA using a T7-oligo(dT) primer, followed by *in vitro* transcription by T7 RNA polymerase with incorporation of Cy3- and Cy5-labeled CTP. 825 ng of the universal reference and confrontation samples labeled once with each of two fluorescent dyes were pooled and fragmented at 60 °C for 30 minutes in a 30 µl reaction following the manufacturers’ instructions. 30 µl of 2x GEx Agilent hybridization buffer HI-RPM (Gene expression hybridization kit, Agilent # 5188–5242) was then added to the fragmentation mixture and hybridized to Agilent SurePrint Custom GE 4 × 180 microarrays (Agilent #G4862A-037207) for 17 hours at 67 °C in a Tecan HS 4800 Pro Hybridization Station (Tecan, Männedorf, Switzerland)^62^. Each confrontation sample was hybridized against the universal reference sample in four replicates. In total, 16 microarrays were used in dye swap configuration to minimize dye bias. After hybridization, microarrays were washed 1 minute at room temperature with GE Wash Buffer 1 (Agilent # 5188–5325) and 1 minute with GE Agilent Wash buffer 2 (Agilent # 5188–5326). Microarrays were scanned with an Agilent G2505C scanner at a resolution of 5 µm in double pass mode, with both green and red channels at 100% laser power, to yield 20 bit tiff images. The scanned images were analyzed with Feature Extraction Software version 10.10.1.1 (Agilent) using default parameters.

All analyses were performed using the statistical environment R (www.r-project.org) and Bioconductor libraries (www.bioconductor.org). Differential expression calls were made using an empirical Bayes regularized *t*-test in the Bioconductor *limma* framework after conservative Benjamini-Yekutieli correction for multiple testing for strong control of the false discovery rate (FDR) to *q* < 5%.

Gpr1-dependent mycoparasitism-relevant genes were identified as follows: First, the strain specific prey responses were assessed by comparing gene expression induced by the prey with gene expression levels under non-mycoparasitic conditions (self-confrontation control). Specifically, we determined the *T*. *atroviride* WT strain response (WT-resp = “WT + prey” *versus* “WT + WT”) as well as the *gpr1* mutant response (*gpr1*-resp = “*gpr1* + prey” *versus “gpr1* + *gpr1”*). Subsequently, the WT and mutant responses were compared resulting in a set of genes dependent on Gpr1 under mycoparasitic conditions (WT-resp *versus gpr1-*resp). This second-level comparison was directly computed as a specific contrast in the linear model, allowing statistical tests of significances to combine evidence across samples.

These tests were both performed for gene transcripts and for functional groups assigned by the FunCat algorithm^63^ employing a Bayesian approach that takes the hierarchical structure of the FunCat categories into account^64^. Improving on traditional tests^65^, the Bayesian approach mitigates issues of noise for Gene Ontology nodes that map to a small numbers of genes, allowing a transfer of knowledge from parent to children nodes in the Gene Ontology graph.

In order to maximize sensitive recall of relevant candidate genes that are robust under method choice, we combined evidence from a range of conservative and more aggressive normalization approaches in rank-product meta-analysis. In line with practice in the field and observations in large-scale benchmark studies^66,67^, we here focus on candidate genes with a large average effect strength, employing a conservative threshold of |aLog_2_FC| > 1.5.

Based on database gene annotation, every gene implicated in the transcriptome analysis was manually curated, and Pfam and InterPro functional protein domains were identified using the NCBI Basic Local Alignment Search Tool (BLAST) for proteins (https://blast.ncbi.nlm.nih.gov/Blast.cgi) and InterproScan sequence search (https://www.ebi.ac.uk/interpro/search/sequence-search), employing the online tools current at the time with default parameters (September 1, 2016). Prediction of transmembrane helices and signal peptide cleavage sites was performed using TMHMM Server v. 2.0^68^ and SignalP 4.1^69^ with default parameters as hosted at CBS at the time (December 14, 2016). Table S1 describes the differently regulated genes. Probe level measurement data are available on request and expression profiles are under submission to Array Express.

### Growth rate, germination and dual confrontation assays

For assessing the growth rate of *T*. *atroviride* WT and mutants, the strains were cultivated on PDA plates at 25 °C in darkness. Radial growth was measured every 24 hours for 10 days. For the analysis of conidial germination, 100 ml Erlenmeyer flasks containing either 50 ml of PDB or minimal synthetic medium (pH 5.5, 2 g/L KH_2_PO_4_, 1.4 g/L (NH_4_)SO_4_, 0.3 g/L CaCl_2_ × 2H_2_O, 0.3 g/L MgSO_4_ × 7H_2_O, 0.05% peptone, 1% glucose, 0.5 g/L FeSO_4_ × 7H_2_O, 0.2 g/L ZnSO_4_ × 7H_2_O, 0.2 g/L MnSO_4_ × 7H_2_O) were inoculated with 10^5^ spores /ml medium (final concentration). After 12 hours of incubation at 25 °C and 200 rpm, conidial germination was investigated using a Thoma chamber (0.100 mm depth Profondeur, 0.0025 mm²) and a Nikon optiphot-2 microscope.

Confrontation assays of *T*. *atroviride* against *R*. *solani* were performed as described above. Plates were incubated for seven days and the growth of *Trichoderma* against itself was set up as a zero inhibition rate. The growth inhibition of *R*. *solani* was calculated implying that the distance between the plugs of both fungi is 100%. Growth inhibition of the prey fungus was calculated as a percentage of *Trichoderma* growth corrected for the growth against itself.

### Plasmid constructions and genetic manipulation of *T*. *atroviride*

*T*. *atroviride* gene deletion strains were created by the split marker technique using the *E*. *coli hph* gene under control of the *A*. *nidulans gpd1* promoter and *trpC* terminator as selection marker^60^. All rounds of PCR amplification and fusion reactions were performed by double joint-PCR^70^ using primers listed in Table S3. Flanking regions (~1500 bp upstream and downstream of the *sfp2* coding region) were amplified from *T*. *atroviride* genomic DNA using primers promoter sur7fw/sur7Rv (upstream) and terminator sur7fw/surRv (downstream). *hph* split marker fragments^72^ were amplified from pBluescript II KS (−)_hph plasmid^71^. 3 µg of each DNA fragment were used for protoplast-mediated fungal transformation. Transformants were purified by three rounds of single spore isolation and deletion of the *sfp2* gene (Ta156007) confirmed by PCR genotyping using the primer pair P5Fw/P5Rv (Table S3), located in the flanking regions, outside of the integrated deletion cassette. In order to create *sfp2* overexpressing mutants, the gene was amplified from genomic DNA and cloned downstream of the constitutively active *T*. *reesei tef1* promoter (GenBank Accession No. Z23012.1).

For complementation of the Δ*sfp2* mutant, a vector expressing s*fp2* C-terminally fused to mEGFP (from pEGFP-N1, GenBank Accession No. U55762.1) and expressed under control of the *T*. *reesei pki1* promoter^73^ was integrated into its genome and transformants selected based on their nourseothricin resistance mediated by the *nat1* gene^74^. The P*pki1*::*sfp2*-mEGFP*::Tsfp2* construct was also introduced into *T*. *atroviride* wild type background. The same approach was used to generate strains expressing Gpr1-mEGFP fusion proteins from a P*pki1*::*gpr1-mEGFP*::T*gpr1* cassette. All primers used for cloning, transformation and genotypic verification are listed in Table S3. Transformants growing in the presence of 300 µg/ml nourseothricin sulfate (Jena Bioscience) were purified by three rounds of single spore isolation. Genomic integration of the respective expression cassettes was confirmed by genotyping PCR, and expression of fluorescent Sfp2-mEGFP and Gpr1-mEGFP fusion proteins was verified by live-cell imaging microscopy.

### Transcript analysis by RT-qPCR

5 µg of DNase I- treated RNA were reverse transcribed with the RevertAid H Minus First Strand cDNA Synthesis Kit (Thermo Scientific) according to the manufacturer’s protocol with a combination of the provided oligo(dT) and random hexamer primers. All real-time PCR experiments were performed in triplicates on a Bio-Rad (Hercules, CA) iCycler IQ. 25 μl assays with IQ SYBR Green Supermix (Bio-Rad, Hercules, CA), standard MgCl_2_ concentration (3 mM), and a final primer concentration of 100 nM each were used. Primer sequences are provided in Table S4. The amplification protocol consisted of an initial denaturation step (2 min at 95 °C) followed by 40 cycles of denaturation (5 sec at 95 °C), annealing (20 sec, for Tm see Table S4) and extension (65 °C for 10 sec) followed by a melting curve analysis. Determination of qPCR efficiency was performed using triplicate reactions from a dilution series (1, 0.1, 10^−2^ and 10^−3^) of cDNA. Amplification efficiency was calculated from the given slopes in the IQ5 Optical system Software v2.0. Expression ratios were calculated using Livak test model^75^ with *sar1* as reference gene^7^ and are given in Table S4.

In addition to RT-qPCR analyses *tac2*, *tac6*, *ech42* (*chi18-5*) and *tef1* listed in Gruber *et al*.^31^ were used for semi-quantitative RT-PCR. The *tef1* gene was employed as reference whereas *ech42* was a positive control for the induction of the chitin degradation machinery. The amplification protocol included an initial denaturation for 30 sec at 98 °C followed by 26 cycles of denaturation (1 min at 98 °C), annealing (1 min, for Tm see Table S4) and extension (72 °C for 1 min). Final extension was performed for 5 min at 72 °C.

### Phylogenetic analysis

We examined the genomic gene content and position of *Trichoderma* Sur7 family proteins in the phylogenetic tree based on their sequence similarity to *S*. *cerevisiae* and *A*. *nidulans* Sur7 family members^15^. Protein sequences were obtained by BLAST from each of the *Trichoderma* spp. and *A*. *nidulans* genomes available on the JGI portal (http://genome.jgi.doe.gov), NCBI (https://www.ncbi.nlm.nih.gov/genome/), and from http://www.yeastgenome.org/ for *S*. *cerevisiae*. Predicted amino acid sequences were aligned by the MUSCLE analysis tool^76^ and the Dayhoff amino acid substitution model was applied for Bayesian analysis. Metropolis-Coupled Markov Chain Monte Carlo (MCMCMC) sampling was performed using MrBayes v3.2.5 with two simultaneous runs of four incrementally heated chains that performed three million generations in total. Trees were summarized after dropping the first 25% of the trees (burn-in). Two completely independent analyses starting from different random trees were carried out. Bayesian posterior probabilities (PP) were obtained from the 50% majority-rule consensus of trees sampled every 100 generations after burn-in. PP values lower than 0.95 were not considered significant and are not shown in the resulting cladogram.

### Confocal laser scanning microscopy

Strains expressing either Gpr1-mEGFP or Sfp2-mEGFP were grown on PDA for 48 h at 25 °C and 12 h dark/light cycles. Agar blocks of about 1 cm² carrying the mycelium were cut out and inverted onto a glass cover slide. Images were recorded on a Nikon C1 confocal laser-scanning unit mounted on a Nikon Eclipse TE2000-E inverted microscope base. EGFP-labeled proteins were excited with the 488 nm laser line of an argon ion laser, and emitted fluorescence light separated by a Nikon MHX40500b/C100332 filter cube was detected with a photomultiplier tube within the range of 500–530 nm. A Nikon Plan Apo VC 60×/1.2 water immersion objective lens was used. Bright-field images were captured simultaneously with a Nikon C1-TD transmitted light detector mounted behind the condenser turret.

Visualization of Δ*sfp2* and WT cell walls was performed on the same Nikon C1 LSCM system using the following cell wall stains: Calcofluor White (CFW) M2R (Sigma #F3543) at a final concentration of 2 µM to non-specifically label β-1,4-glucans including chitin, Solophenyl Flavine (SPF) 7GFE 500 (Ciba #1485385V6) at a final concentration of 20 µM to selectively label polysaccharides containing β-1,4 linkages, and Congo Red (CR) (Sigma #C6767) at a final concentration of 100 µM to very specifically label α- and β-chitin. All dyes were left to incubate on the cells for at least 15 min before imaging. CFW and SPF were excited with 405 nm light from a blue diode and emission light was detected between 430–470 nm. CR was excited with 543 nm from a HeNe laser and emission light was detected between 580–620 nm. Measurements of distances between septa and of hyphal diameters were performed with the corresponding plugins of the MacBiophotonics ImageJ work package available at (https://www.macbiophotonics.ca/software.htm), and statistically evaluated using the ggplot2 package of R (https://www.r-project.org/).

To assess endocytosis in growing hyphae, the membrane-selective fluorescent dye FM4-64 (#T3166, ThermoFisher Scientific) was used at a final concentration of 1.67 µM and imaged on a Leica LSM SP5 confocal microscope fitted with a Leica 63x/1.3 N.A. objective lens. Fluorophore excitation was achieved with the 488 nm line of an Argon laser, and emission light was detected with a PMT between 600 nm and 700 nm. For time course recordings, images were taken with a frame rate of 1 min^−1^ over the desired period for up to 150 min. To evaluate differences in endocytosis rates between the tested strains, we used three key stages of the dye uptake process as readily discernable visual markers (stage I: plasma membrane staining, stage II: appearance of endocytic vesicles and stage III: complete dye internalization) and recorded the time at which these phenotypes first appeared in each strain after dye addition.

In all cases, excitation laser intensity and laser dwell time during image acquisition were kept to a minimum to reduce photobleaching and phototoxic effects while providing a sufficient signal-to-noise ratio for quantitative image analysis. All images were recorded with a maximum resolution of 1024 × 1024 pixels and saved as TIFF. Apart from display range adjustments and cropping using the ImageJ software platform (http://rsb.info.nih.gov/ij/), images were not subjected to further manipulation.

## Electronic supplementary material

Supplementary information

Supplementary Dataset 1

## Data Availability

All data generated or analysed during this study are included in this published article and its Supplementary information files and are available on reasonable request.
