# Supplementary material for: The Gpr1-regulated Sur7 family protein Sfp2 is required for hyphal growth and cell wall stability in the mycoparasite Trichoderma atroviride
Source: Sci Rep. 2018 Aug 13;8:12064. doi: 10.1038/s41598-018-30500-y (PMC6089919; doi:10.1038/s41598-018-30500-y)
Supplement: Supplementary file 1 — Supplementary information [file 41598_2018_30500_MOESM1_ESM.pdf]

# The Gpr1-regulated Sur7 family protein Sfp2 is required for hyphal growth and cell wall stability in the mycoparasite *Trichoderma atroviride*

Lea Atanasova[1$], Sabine Gruber[1*], Alexander Lichius[1*], Theresa Radebner[2], Leoni Abendstein[1], Martin Münsterkötter[3,4], Nancy Stralis-Pavese[5], Paweł P. Łabaj[5], David P. Kreil[5] and Susanne Zeilinger[1,2§]

*1) Institute of Microbiology, University of Innsbruck, Innsbruck, Austria*
*2) Institute of Chemical, Environmental & Biological Engineering, TU Wien, Vienna, Austria*
*3) MIPS - Institute of Bioinformatics and Systems Biology, Helmholtz Zentrum München, Germany*
*4) Functional Genomics and Bioinformatics, Sopron University, Sopron Hungary*
*5) Chair of Bioinformatics, Boku University Vienna, Vienna, Austria*
*[$] Current address: Institute of Food Technology, University of Natural Resources and Life Sciences (BOKU), Vienna, Austria*

[§]corresponding author: Susanne Zeilinger, Susanne.zeilinger@uibk.ac.at

*these authors contributed equally to the manuscript

# Supplementary Data

Supplementary Table 1: Differently regulated genes

Supplementary Table 2: FunCat categories assigned to differently regulated genes

Supplementary Table 3: Oligonucleotides used

Supplementary Table 4: Primers for gene expression analysis

Supplementary Table 5: RT-qPCR quantification of selected genes

Supplementary Figure 1: Genomic environment of the *gpr1* gene

Supplementary Figure 2: Semi-quantitative expression analysis of chitinase genes

**Table S1:** List of *T. atroviride* genes whose transcription is significantly differently regulated comparing the responses of the *gpr1*-silenced mutant *gpr1* sil-8 and the WT to *R. solani*. The robustness of the combined evidence from multiple analyses is indicated by the aggregate rank (aRank; the lower the rank the more robust evidence is provided by the data). aLogFC (WT) and aLogFC (*gpr1*-si) show the average log fold-changes of the respective strains' response to the prey; aLogFC (WT-*gpr1*-si) represents the aLogFC of the *gpr1*-si mutants' response subtracted from the WT response (for details see the Methods section). Filtered models correspond to the protein IDs of the filtered models in the JGI DOE *T. atroviride* genome database.

Please see file "Supplementary Tables S1 to S5.xls"

**Table S2:** List of FunCat categories assigned to transcripts that were significantly differently regulated comparing the responses of the *gpr1* sil-8 mutant and the WT to *R. solani*. The most robustly identified categories with the lowest aggregate rank (aRank) are listed first. Aggregate enrichment score (r) values close to 1 and 0 annotate overrepresented and underrepresented categories, respectively. Aggregate direction (aDir) values close to 1 or -1 show good agreement across analysis variants with over- or underrepresentation of categories, whereas values close or equal to 0 mark poor or no agreement across the analyses, respectively. Aggregate significance (aSig) represents the percentage of analyses that inferred an under- or overrepresentation of categories with a probability larger than 90%. The yellow highlighted FunCat codes mark the highest level annotation categories. The functional categories tested correspond to the functional classification catalogue (FunCat) version 2.1 (http://mips.helmholtz-muenchen.de/funcatDB/) slightly adapted to version 2.0 as listed in Ruepp et al.[61].

Please see file "Supplementary Tables S1 to S5.xls"

**Table S3**: Oligonucleotides used for cloning, genotypic verification, sequencing and generation of transformation cassettes.

Please see file "Supplementary Tables S1 to S5.xls"

**Table S4**: qPCR and RT-PCR primers for gene expression analysis used in this study.

| Gene | Tm [°C] | Primers (5'→ 3') fw | rev | PE |
|---|---|---|---|---|
| *sar1* | 60 | CTCGACAATGCCGGAAAGACC | TTGCCAAGGATGACAAAGGGG | 1 |
| *tef1* | 58.5 | TACTATGTCACCGTCATTG | CAGCGATAATCAGGATA | 1 |
| actin | 60 | GCACGGAATCGCTCGTTGC | TTCTCCACCACCGCCAAGC | 1 |
| *ech42* | 60 | CGCAACTTCCAGCCTCAGAACC | TCAATACCATCGAAACCCCAGTCC | 1 |
| chs1 | 57 | ACTCGGACCTCAATGGAA | GGCGGCTATGAAGTAACG | 0.9 |
| chs2 | 57 | TCCGACCATTCATTCTTC | CCAGACTTGTAATCAGGAT | 1 |
| tac2 | 55 | GCCCTCGTGCTCCATCAG | GGTCTCGTAGTTGCCGGG | 1 |
| tac6 | 55 | CGGGACTTATGGTTTGGGCGG | CGAACGGTCCAGATGCGGG | 1 |
| *hsp70* | 58 | GGTGCTACAGTCAAGTAA | CCATAGGGAAAGAAACAAAG | 1 |
| *tef2* | 58 | AAGAAGTGGACCAAGAAC | TCATAACAGCAGAGAAGAT | 1 |
| *gh3* | 58 | TATGAGAGCATCCTATCC | AACACTAATGTAGCCTTC | 1 |
| *coA* | 60 | ATGTCGTTATTGCTTCTT | CATTAAATGTGGAGGGTTA | 1 |
| *sur4* | 55.7 | TACACTTACTACACTCTG | AATGTATGTGATGAAGGA | 0.96 |

**Table S5**: Real Time PCR quantification of selected genes for verification of microarray data.

| Gene | Gene name | Protein ID | Microarray data [log 2 (ratio)] | | qPCR verification Relative expression | |
|---|---|---|---|---|---|---|
| | | | WT | *gpr1*-si | WT | *gpr1*-si |
| Putative protein of HSP70 family | *hsp70* | 284614 | -2.080 | 3.780 | 0.085 | 1.853 |
| Transcription elongation factor 2 | *tef2* | 301275 | -4.020 | 1.350 | 0.026 | 1.363 |
| GH3 with Fibronectin type III-like domain | *gh3* | 302027 | -3.630 | 0.980 | 0.003 | 14.806 |
| 3-hydroxyacyl-CoA dehydrogenase | *coA* | 80898 | 3.230 | 0.00006 | 2.580 | 0.239 |
| Putative SUR4 fatty acid elongase | *sur4* | 301574 | 1.890 | 0.080 | 4.448 | 0.127 |

**Figure S1:** The genomic environment of the *gpr1* gene in *T. atroviride* (TA), *T. virens* (TV) and *T. reesei* (TR) is conserved and includes *sfp2*, a gene coding for a Sur7 family protein with four transmembrane domains.

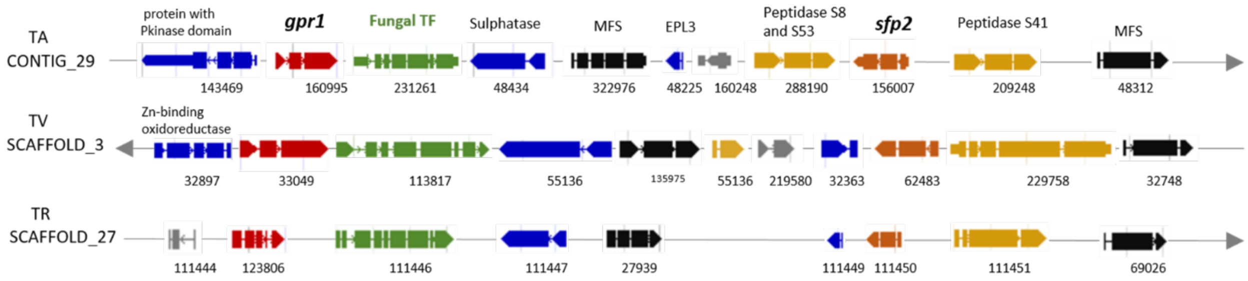

**Figure S2:** Semi-quantitative expression analysis of chitinase genes (A) *tac2* (Ta217701), (B) *tac6* (Ta214040) and (C) *ech42* (Ta131598) in *T. atroviride* WT, *sfp2* and *gpr1-si* mutants cultivated in liquid PDB medium with and without Congo Red as cell wall stress inducer. (D) The translation elongation factor1–encoding gene *tef1* was used as a control[31].

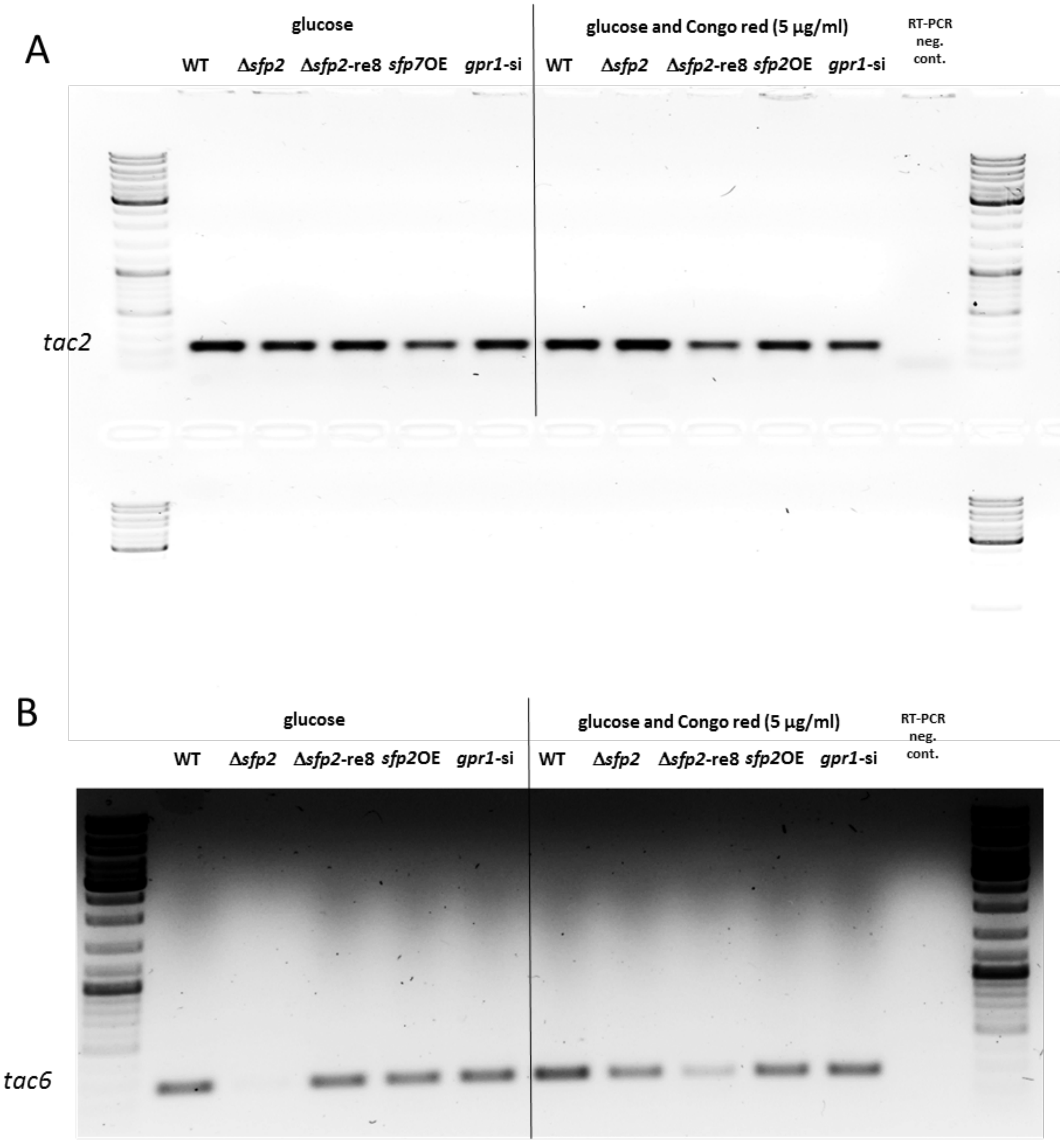

C

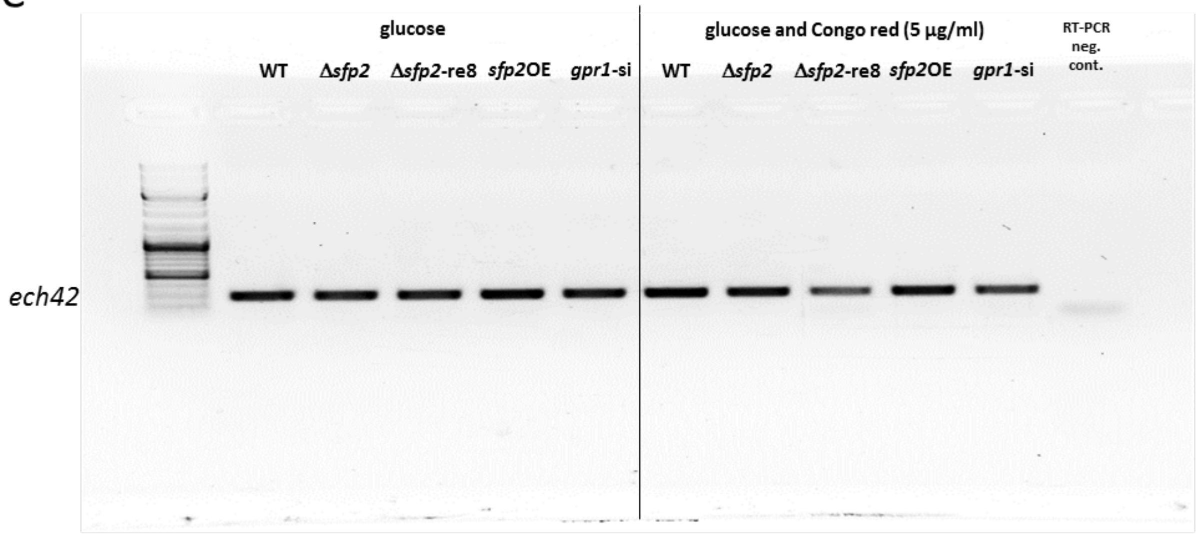
glucose
glucose and Congo red (5 µg/ml)
RT-PCR neg. cont.
WT
Δ*sfp2*
Δ*sfp2*-re8
*sfp2*OE
*gpr1*-si
WT
Δ*sfp2*
Δ*sfp2*-re8
*sfp2*OE
*gpr1*-si
*ech42*

D

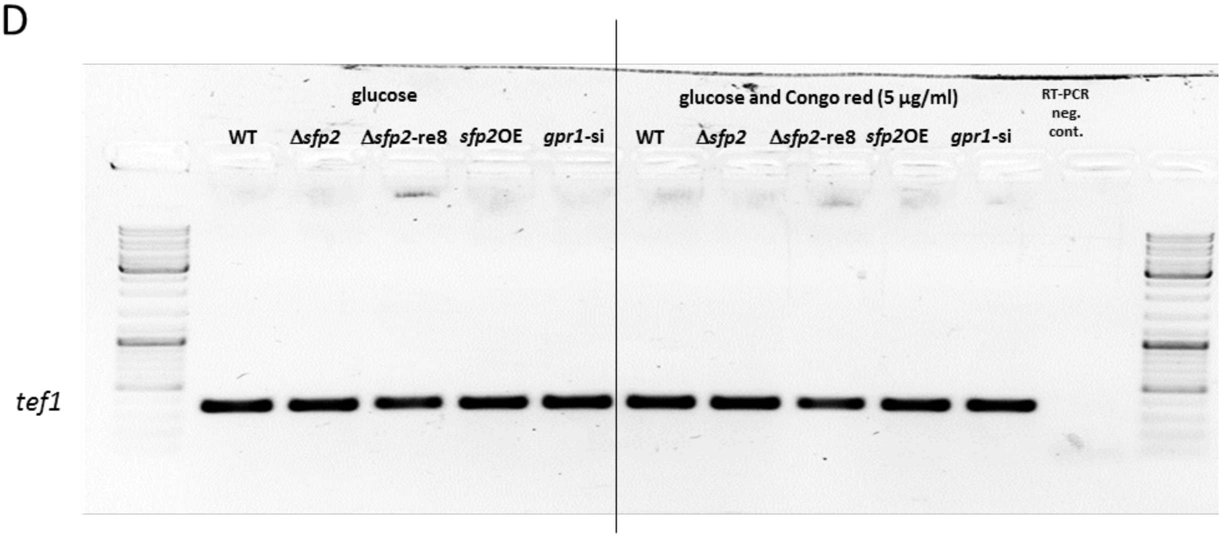
glucose
glucose and Congo red (5 µg/ml)
RT-PCR neg. cont.
WT
Δ*sfp2*
Δ*sfp2*-re8
*sfp2*OE
*gpr1*-si
WT
Δ*sfp2*
Δ*sfp2*-re8
*sfp2*OE
*gpr1*-si
*tef1*
